# The Role of Genetics in Advancing Precision Medicine for Alzheimer’s Disease—A Narrative Review

**DOI:** 10.3389/fmed.2018.00108

**Published:** 2018-04-24

**Authors:** Yun Freudenberg-Hua, Wentian Li, Peter Davies

**Affiliations:** ^1^Litwin-Zucker Center for the study of Alzheimer’s Disease, The Feinstein Institute for Medical Research, Northwell Health, Manhasset, NY, United States; ^2^Division of Geriatric Psychiatry, Zucker Hillside Hospital, Northwell Health, Glen Oaks, NY, United States; ^3^Robert S Boas Center for Genomics and Human Genetics, The Feinstein Institute for Medical Research, Northwell Health, Manhasset, NY, United States

**Keywords:** Alzheimer’s disease, genetics, genomics, risk factors, risk variants, precision medicine, genome sequencing

## Abstract

Alzheimer’s disease (AD) is the most common type of dementia, which has a substantial genetic component. AD affects predominantly older people. Accordingly, the prevalence of dementia has been rising as the population ages. To date, there are no effective interventions that can cure or halt the progression of AD. The only available treatments are the management of certain symptoms and consequences of dementia. The current state-of-the-art medical care for AD comprises three simple principles: prevent the preventable, achieve early diagnosis, and manage the manageable symptoms. This review provides a summary of the current state of knowledge of risk factors for AD, biological diagnostic testing, and prospects for treatment. Special emphasis is given to recent advances in genetics of AD and the way genomic data may support prevention, early intervention, and development of effective pharmacological treatments. Mutations in the *APP, PSEN1*, and *PSEN2* genes cause early onset Alzheimer’s disease (EOAD) that follows a Mendelian inheritance pattern. For late onset Alzheimer’s disease (LOAD), *APOE4* was identified as a major risk allele more than two decades ago. Population-based genome-wide association studies of late onset AD have now additionally identified common variants at roughly 30 genetic loci. Furthermore, rare variants (allele frequency <1%) that influence the risk for LOAD have been identified in several genes. These genetic advances have broadened our insights into the biological underpinnings of AD. Moreover, the known genetic risk variants could be used to identify presymptomatic individuals at risk for AD and support diagnostic assessment of symptomatic subjects. Genetic knowledge may also facilitate precision medicine. The goal of precision medicine is to use biological knowledge and other health information to predict individual disease risk, understand disease etiology, identify disease subcategories, improve diagnosis, and provide personalized treatment strategies. We discuss the potential role of genetics in advancing precision medicine for AD along with its ethical challenges. We outline strategies to implement genomics into translational clinical research that will not only improve accuracy of dementia diagnosis, thus enabling more personalized treatment strategies, but may also speed up the discovery of novel drugs and interventions.

## Introduction

Alzheimer’s disease (AD) is the most common form of dementia (1) accounting for 60–80% of dementia diagnosis and affects nearly 50 million people worldwide (2). The worldwide number of affected individuals is expected to reach 66 million by 2030, and 131 million by 2050 (3) as the number of older adults increases. One in 10 people over age 65 and every third person over age 85 in the US has a diagnosis of AD (4). The global financial toll of dementia was estimated to be 818 billion US dollars in 2015, an increase of 35% since 2010 and this cost is expected to further rise together with the prevalence of AD (2). The majority of the costs are related to family and social care of patients, rather than medical care. About 5% of all AD patients show cognitive symptoms before age 65 and are classified as early onset Alzheimer’s disease (EOAD) (5). Patients showing clinical symptoms after age 65 are classified as having late onset Alzheimer’s disease (LOAD). Here, we provide a summary of the clinical, neuropathological, fluid, and imaging biomarkers of AD along with a more comprehensive review of genetic findings in both Mendelian and sporadic forms of AD. We discuss how genetic analysis as applied in Mendelian randomization (MR) may be helpful in validating causality of modifiable risk factors that could advance preventive measures. Moreover, genetic data may be useful to facilitate precision medicine. The goal of precision medicine is to integrate clinical, genetic, and life style data to enable clinicians to efficiently and accurately predict the most appropriate course of action for a patient (6). We emphasize the ways genetics may facilitate precision medicine in AD: (1) identifying at risk individuals through risk prediction, (2) improving diagnostic precision, and (3) expediting the discovery of targetable disease mechanisms for drug development. Due to the large number of published articles in biomedical research of AD, we refer to more recent comprehensive reviews written by domain experts and supplement these with other findings.

## Literature Selection

Our goal of writing this narrative review (7) is to discuss how genetics may not only advance basic research on disease mechanisms but also play a role in facilitating precision medicine in AD. We provide summaries about clinical and neuropathological features, research on imaging and fluid biomarkers, as well as modifiable risk factors of AD by referring to high-quality recent systematic reviews and meta-analyses. Unpublished or original data, submitted manuscripts, or personal communications are excluded. More recent scientifically rigorous and high-impact studies on these topics that were found in the PubMed database, but not previously reviewed and those having a historical impact were also included. Over the past 20 years, our understanding about genetic research has expanded together with the rapidly advancing technology. The quality requirement for genetic studies has also evolved from candidate gene approaches, which were often criticized for producing inconsistent and non-replicable results (8), to more thoroughly conducted and well-powered genome-wide studies (9). We included publications of the Mendelian AD genes as well as publications that were referred and curated by the National Human Genome Research Institute-European Bioinformatics Institute (NHGRI-EBI) Catalog of published genome-wide association studies (GWAS Catalog) (10). In addition, we included high-quality association studies reporting rare variants that meet the “analytically rigorous” criteria for GWAS (9) or are otherwise statistically thorough.

## Clinical Features of AD

In 1906, the German psychiatrist Alois Alzheimer first described the clinical features of an early-onset case of AD with its pathognomonic hallmarks—extracellular amyloid (neuritic) plaques and intracellular neurofibrillary tangles (NFT) (11). Patients typically show an insidious onset and continuous cognitive decline, which typically starts with an amnestic presentation with impaired ability to remember new information. The cognitive decline may further affect language, reasoning, executive function, visuospatial abilities, and the illness is often accompanied by personality and behavioral changes that affect the social function of the patient. In an advanced disease stage, patients are completely dependent on their caregivers for daily functioning such as getting dressed, toileting, mobility, and eating. The NINCDS-ADRDA criteria for diagnosing possible and probable AD are being widely used (12) and have a sensitivity and specificity of ~70% for distinguishing between AD patients and people without dementia. However, they were less accurate distinguishing between different types of dementias (13, 14). The median survival time of patients from the symptom onset is reported to be 9 years (15).

## Neuropathology of AD

Over many years, definitive diagnosis of AD could only be made by the “gold standard” of postmortem neuropathological examination, using a combination of CERAD score for neuritic plaques containing amyloid beta (Aβ) (16) together with Braak staging of NFT consisting of abnormally hyperphosphorylated tau (17). This had been defined in the National Institute on Aging (NIA)-Reagan Criteria (18). However, only half of the brains of patients with the clinical diagnosis of probable AD showed “pure” AD pathology (19). In 2011, the NIA and the Alzheimer’s Association (AA) revised the diagnostic criteria aimed at integrating the advances of imaging and cerebrospinal fluid (CSF) biomarkers to model the three stages of AD that include preclinical stage, mild cognitive impairment, and dementia (12, 20–22). The updated criteria are now used in AD research and ongoing efforts exist to refine these criteria (23). It is important to emphasize that Aβ deposits have not been proven to be causal for late onset AD. In addition to Aβ and NFT, other neuropathological features such as TDP-43 immunoreactive inclusions and Lewy bodies may coexist, along with findings like cerebral amyloid angiopathy, cerebrovascular disease, and hippocampal sclerosis (19, 24–27). It is important to note that AD pathologies were also found in nearly all brain autopsies of cognitively normal individuals above age 80, even among those considered as high-cognitive performers (28, 29). Although some cognitively normal elderly had severe AD pathologies, as a group, they showed less severe AD pathologies than dementia patients. Signs of vascular injuries ranged from 32% among high cognitive performers to 64% in late dementia subjects.

## Imaging and Biomarkers

To provide early and accurate diagnosis of AD, extensive efforts have been made into developing sophisticated methods to assess pathology in the living human brain. However, to date, no test or combination of tests that could accurately diagnose AD is available for broad clinical use outside of AD research centers (4). CSF levels of Aβ42, tau, and hyperphosphorylated tau (ptau) as markers for amyloid, neuronal injury, and tangles, respectively, have been the main fluid biomarkers used in AD research (30, 31). In CSF of AD patients, a decreased level of Aβ42 has been consistently found (32), whereas the concentrations of tau and ptau are increased (31). Levels of CSF tau and ptau, but not Aβ42, were found to correlate with brain atrophy in AD (33). Interestingly, a reduction of CSF Aβ42 had been shown to correlate with brain atrophy in non-demented elderly indicating a potential preclinical stage (33).

Unaddressed problems preventing broad clinical utility of biomarkers include incomplete clinical validity, inconsistent predictive value, and assay variability (34). The consensus from experts in the field of biomarkers concludes that CSF AD biomarkers may be used alongside clinical measures to identify or exclude AD as an underlying cause particularly in uncertain and atypical clinical presentations (35).

In parallel to CSF biomarkers, major advances were made to measure Aβ and tau deposits *in vivo* with help of brain imaging. Using a combination of 18-fluoro-deoxyglucose-positron emission tomography (PET), which measures cerebral glucose metabolism, and Pittsburgh compound B (PIB) PET measuring the Aβ deposition along with CSF biomarkers, it was demonstrated that subjects with known Mendelian AD mutations have CSF Aβ changes, brain amyloidosis, tauopathy, brain atrophy, and decreased glucose metabolism in that same temporal order starting 20 years before the clinical onset of AD (36). More recently, voxel-based hierarchical clustering was applied to cross-sectional flortaucipir PET imaging for ptau and PIB–PET for Aβ in 88 elderly cognitively normal individuals (37). The study identified four tau clusters and four Aβ clusters based on spatial features. It shows that tau clusters map to the temporal lobe and orbitofrontal cortex and expand to parietal and frontal lobes roughly corresponding to Braak tau stages (38), whereas Aβ deposits are dispersed in widespread heteromodal cortex. The finding that tau and Aβ deposits displayed distinguishable locations with some overlap, particularly in the association cortex, suggested that AD is a tau-centered disease with amyloid effects.

## Risk Factors for AD

Currently known risk factors for AD include age, sex, cardiovascular risk factors, metabolic risk factors, sleep apnea, family history, and certain genetic variants (2, 4). Thus, both modifiable and non-modifiable risk factors have been associated with LOAD risk. The non-modifiable factors include sex, aging, and the genetic risk.

## Genetics of Autosomal Dominant AD

A recent systematic review of studies from the US, Europe, India, and China shows that the worldwide proportion of EOAD is around 5% of all AD cases (39). Of note, only 30–60% of EOAD patients have a positive family history for dementia, and about 10–14% have a family history that is consistent with autosomal dominant inheritance (40–42). Thus, in addition to the Mendelian disease presentation of EOAD, a substantial proportion of EOAD cases fall into the category of sporadic and genetically complex disease. For the Mendelian cases, three genes that carry mutations causal for autosomal dominant AD were identified in the 1990s, namely *APP* (43), *PSEN1* (44), and *PSEN2* (45, 46). The *APP* gene encodes amyloid precursor protein which is proteolytically processed into Aβ peptides by β- and γ-secretase. Most pathogenic mutations in *APP* have been reported to either increase Aβ production or influence the ratio of Aβ peptides of different length (e.g., the Aβ42/Aβ40 ratio) resulting in increased self-aggregation (47). Notably, at the same site of a disease causing *APP* mutation that increases APP cleavage, a protective variant leading to a different amino acid change was found that decreases APP cleavage (48). *PSEN1* and *PSEN2* genes encode part of the γ-secretase complex and *PSEN1* accounts for most of the known mutations for autosomal dominant AD. The majority of pathogenic *PSEN1* mutations impair γ-secretase-dependent cleavage of APP and decrease the production of both Aβ42 and Aβ40 (49). These genetic findings in autosomal dominantly inherited EOAD (48, 50) provide strong support for the amyloid hypothesis implicating that Aβ plays an initiating role in AD. A recent review presented a large body of evidence from over 25 years of research supporting the generalizability of amyloid hypothesis (51). However, there are also findings that contradict amyloid being the main driving cause for the more common sporadic manifestations of AD (52). For example, elevated amyloid deposition is frequently found in cognitively normal subjects (28, 53–55) and CSF level of Aβ and Aβ imaging with PIB–PET do not correlate with cognitive decline (56). Furthermore, Aβ production is reduced by most *PSEN1* mutations (49). The anatomic and temporal discordance between Aβ pathology, tau aggregation, and neurodegeneration has led to the postulation of Aβ being an initiator of a complex cascade that ends in tau-medicated neurodegeneration (57).

## Genetics of LOAD

**Figure 1:**
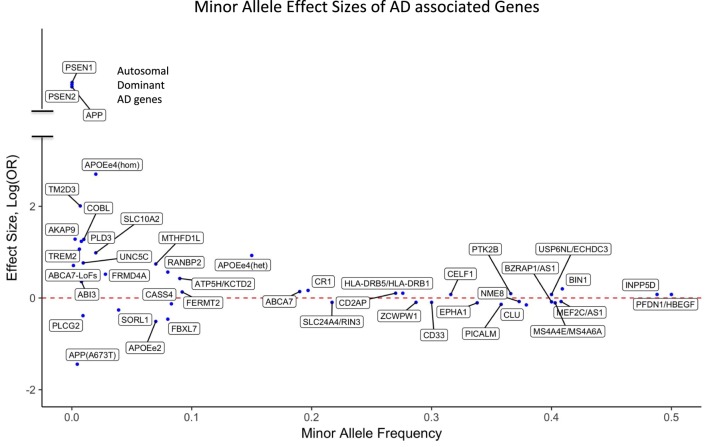
Effect sizes of AD associated variants for the respective minor alleles. The red dotted line indicates OR = 1 [log(OR) = 0]. Minor alleles with log(OR) above the line are risk alleles and below the line are protective. Abbreviations: APOEe4(hom), homozygosity for the *APOE4* allele; APOEe4(het), heterozygosity for the APOE4 allele; ABCA7-LoFs, aggregated effects of loss-of-function variants in ABCA7; OR, odds ratio; AD, Alzheimer’s disease.

For the majority of AD patients, no known causal genetic mutations have been identified. LOAD as well as many cases of EOAD are genetically complex and have multifactorial causes, which is similar to other chronic common diseases. A large population-based twin study estimated that genetic factors contribute 58–79% of etiologic role for LOAD (58). More than 20 years ago, *APOE4* (also called *APOE* ε4) allele of the *APOE* gene has been identified as a major genetic risk factor for LOAD (59, 60). The *APOE* gene has two missense variants at amino acid residues 112 and 158 leading to three common haplotypes, which are typically referred to as APOE alleles ε2 (Cys and Cys), ε3 (Cys and Arg), and ε4 (Arg and Arg). Among Caucasians, homozygous ε4 carriers show the highest life time risk for AD (68–91%) (61–64) with an odds ratio (OR) of 11–12.9 compared with homozygous ε3 carriers. Individuals carrying one copy of ε4 have a threefold risk increase for AD compared with people having no ε4 allele, and the ε2 allele is protective against AD (Figure 1). In African-Americans and Hispanic populations the OR of *APOE4* is found to be less pronounced compared to Caucasians. It is important to note that unlike the mutations in autosomal dominant forms of AD, *APOE4* is not a sufficient determinant of AD even in old aged individuals. We have previously reported a homozygous *APOE4* carrier who reached the age of 95 years without overt signs of dementia (65).

*APOE* encodes a lipid carrier Apolipoprotein E (ApoE) that is found both in the periphery and the central nervous system (66). The risk effects of *APOE4* in AD were linked to ApoE’s pleiotropic functions that lead to reduced cholesterol transport, less efficient Aβ clearance and more aggregation, triggering neurotoxicity through Tau phosphorylation, increased brain neuronal activity and atrophy, reduced synaptic plasticity, and greater neuroinflammation. The large body of literature investigating the functional mechanism of ApoE in AD has been recently summarized (67–69). Most recently, ApoE has been shown to affect tau pathogenesis, neuroinflammation, and tau-mediated neurodegeneration independently of amyloid-β pathology in transgenic mice (70).

In addition to the well-established effects of *APOE*, GWAS have identified more than 30 genomic loci that are associated with AD risk. Unlike the *APOE* variants, the majorities of GWAS identified risk variants do not alter the protein sequence and are not necessarily the actual causal variants. Instead, an associated variant may be in linkage disequilibrium with an unidentified causal variant that may alter protein sequence, splicing patterns, or gene expression. In GWAS for LOAD, genes that are located near the associated variants are considered potential risk genes, but further evidences are necessary to support their actual etiological role. As of September 1, 2017, the NHGRI-EBI GWAS Catalog (10) listed 74 published GWAS studies on LOAD. We manually curated this list by merging multiple reports for the same locus into one row (Table 1). It is clear that some gene loci have been replicated by two or more GWAS or meta-analysis. These genes are *BIN1, CD2AP, CLU, CR1, EPHA1, MS4A4E/MS4A6A, PICALM, and TREM2*. The confidence for these genes to be actual AD genes is higher compared with those genes supported by a distant variant in one single study. For example, one association signal on Chromosome 2 was supported by an intergenic variant rs17034806 that is located 200 kb from the gene *RANBP2* (71). In Table 1, if a locus is implicated in more than one association study or is supported by meta-analysis, we show the strongest association signal.

**Table 1:** AD associated loci from the NHGRI-EBI GWAS Catalog.

CHR	Region	Gene locus	Risk allele frequency	*P*-value	Risk allele OR
1	1q32.2	CR1	0.197	6.0E−24	1.18
2	2q13	RANBP2	0.08	4.0E−08	1.76
2	2q14.3	BIN1	0.409	7.0E−44	1.22
2	2q37.1	INPP5D	0.488	3.0E−08	1.08
5	5p15.1	FBXL7	0.92	5.0E−08	1.59
5	5q14.3	MEF2C	0.592	3.0E−08	1.08
5	5q31.3	PFDN1, HBEGF	0.5	7.0E−09	1.08
6	6p21.32	HLA-DRB5, HLA-DRB1	0.276	3.0E−12	1.11
6	6p21.1	TREM2	0.0063	2.0E−12	2.9
6	6p12.3	CD2AP	0.27	9.0E−09	1.11
6	6q25.1	MTHFD1L	0.07	2.0E−10	2.1
7	7p14.1	NME8	0.627	5.0E−09	1.08
7	7p12.1	COBL	0.991	4.0E−08	3.59
7	7q22.1	ZCWPW1	0.713	6.0E−10	1.1
7	7q35	EPHA1	0.662	1.0E−13	1.11
8	8p21.2	PTK2B	0.366	7.0E−14	1.1
8	8p21.1	CLU	0.621	3.0E−25	1.16
10	10p14	USP6NL, ECHDC3	0.4	3.0E−08	1.08
10	10p13	FRMD4A	0.028	1.0E−10	1.68
11	11p11.2	CELF1	0.316	1.0E−08	1.08
11	11q12.2	MS4A4E/MS4A6A	0.597	6.0E−16	1.11
11	11q14.2	PICALM	0.642	9.0E−26	1.15
11	11q24.1	SORL1	0.961	1.0E−14	1.30
13	13q33.1	SLC10A2	0.985	5.0E−08	2.68
14	14q22.1	FERMT2	0.092	8.0E−09	1.14
14	14q32.12	SLC24A4, RIN3	0.783	6.0E−09	1.1
17	17q22	BZRAP1	0.6	4.0E−08	1.09
17	17q25.1	ATP5H, KCTD2	0.09	4.7E−09	1.53
19	19p13.3	ABCA7	0.19	1.0E−15	1.15
19	19q13.32	APOE	0.15	2.0E−157	2.53
19	19q13.41	CD33	0.7	2.0E−09	1.1
20	20q13.31	CASS4	0.917	3.0E−08	1.14

*The database was queried on September 1, 2017 for association studies on AD. If an association locus is reported by multiple GWAS, we merged the results by reporting the most significant P-value for that locus*.

*CHR, chromosome; OR, odds ratio; AD, Alzheimer’s disease; GWAS, genome-wide association studies; NHGRI-EBI, National Human Genome Research Institute-European Bioinformatics Institute*.

Although GWAS have been a powerful method to uncover risk loci in AD, they are less suitable to discover infrequent or rare variants. A recent estimate indicates that only 30.6% of the genetic variance can be explained by known AD single-nucleotide polymorphisms (SNPs), but a sizeable fraction of the unidentified risk variants may be located close to the known risk SNPs, potentially as rare variants (72). Consistent with an important role of rare variants, our investigation using whole genome sequencing (WGS) showed an increased burden of rare loss-of-function variants in immune genes in AD compared with cognitively healthy centenarians (73). Large-scale sequencing, such as whole exome sequencing (WES) and WGS, has already identified new genes that harbor rare variants typically missed by GWAS. Rare variants that increase the risk for AD have been identified in *TREM2* (74, 75), *PLD3* (76, 77), *UNC5C* (78), *AKAP9* (79), *ADAM10* (80), and *ABI3* (81). Moreover, the burden of rare coding variants in risk genes identified by GWAS such as *ABCA7* (82–84) as well as in Mendelian genes for AD had been found to be increased among LOAD patients compared with unaffected general population (85, 86). The potential impact of rare variants in AD is further underscored by rare and low-frequency protective variants such as *APOE2* allele (61, 67), *APP* A673T (48), and *PLGC2* P522R (81). The effect sizes of both GWAS loci and genes harboring reported rare AD-associated variants are presented in Figure 1.

Undoubtedly, the search for rare risk variants with high-effect sizes for LOAD faces many obstacles. First, many studies are underpowered to separate true signals from false-positive associations as tens of thousands of cases and controls are usually required to achieve genome-wide significance level of *P* < 5E−8. Second, allele frequencies of rare variants are more likely to vary between population cohorts of different ethnic backgrounds due to founder effects, making replication studies difficult to conduct. For example, risk allele frequencies in *PLD3* in controls of one cohort may be higher than that of cases in another cohort, while combined result may be nominally significant (77) or not significant at all (87–89). Third, the necessarily small number of carriers of rare variants makes the respective association studies particularly prone to be impacted by factors such as age, *APOE4* carrier status, and different genotyping and sequencing platforms.

## Pathways Implicated by Risk Genes for AD

The established AD associated genes exert pleiotropic functions across many molecular pathways. Several of these pathways stand out by providing insights for the disease mechanisms that may play a role in the etiology of AD (90–92). Major pathways include inflammatory response (*ABCA7, CD33, CLU, CR1, MS4A, INPP5D, TREM2, PLCG2, PTK2B*, and *ABI3*), lipid metabolism (*APOE, CLU, ABCA7*, and *PLCG2*), as well as endocytosis/vesicle-mediated transport (*BIN1, PICALM, CD2AP, EPHA1*, and *SORL1*). Other functional categories include regulation of cell cycle (*RANBP2*), oxidative stress response (*MEF2C*), and axon guidance (*UNC5C*).

A role of innate immunity and inflammation in AD etiology is independently supported by a large body of functional evidence (93–95). Among the risk genes from the immune pathways, *TREM2* stands out with its high effect-size of AD risk (74, 75). *TREM2* stands for triggering receptor expressed on myeloid cells 2, a single-transmembrane protein expressed by monocytic myeloid cells. Both ApoE and Clusterin (encoded by *CLU*) are extracellular chaperons that prevent protein aggregation. In addition, both bind to the microglial receptor TREM2 and thus may promote uptake of Aβ by microglia (96). Studies on animal and human brains indicated that the TREM2 risk variant p.R47H impairs TREM2 detection of lipid ligands leading to microglia dysfunction (97, 98). In addition to *TREM2*, the two newly identified AD risk genes *ABI3* and *PLCG2* are highly expressed in microglia as well (81).

**Figure 2:**
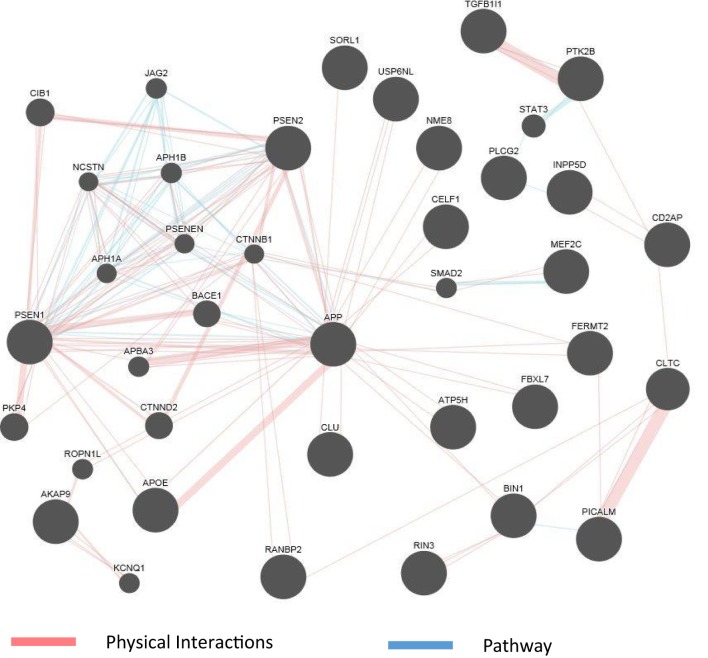
GeneMania network for physical interaction (PI) and pathway. An example of GeneMANIA network when only the PI and pathway links are used. Alzheimer’s disease genes from input list are presented as large black circles, and other genes deemed to be associated with the cluster are small black circles. Genes not linked to the main cluster are discarded.

The abundance of genomics data in the public domain can be utilized not only to confirm the known connections among AD genes but also to reveal potentially new genes involved in the disease. Figure 2 shows an example of a network representation of AD genes by the GeneMANIA software tool (99). AD genes, as well as other genes deemed to be appropriate by the program, can be linked by criteria such as coexpression, physical interaction (PI) studies, or being part of the same pathway. Figure 2 shows an example of visualization of PI and pathways of a subset of AD genes reviewed in this article. The known high impact AD genes (*APP, APOE, PSEN2*, and *PSEN1*) are also highly connected genes. New genes introduced by this program may be further investigated as potential candidate genes for AD. As the computational methods to integrate larger biological data sets continue to improve and be refined, known risk genes may predict gene sets (100) and pathways that can be targeted by drugs.

## Polygenic Risk Scores

Because many AD risk SNPs are common variants, every individual necessarily inherits multiple such risk alleles. A polygenic risks score (PRS) (101) can be calculated based on the number of common genetic risk factors present in an individual’s genome, which may be used as predictor for AD risk (102, 103). Using the area under the curve receiver operator characteristic method, PRS may capture nearly all of the genetic liability from common risk variants for AD. However, the efficacy of a genetic predictor is dependent on prevalence and heritability of a disease (104). In AD, the prevalence is highly dependent on age. For the younger age group (65–74) PRS profile captured 90% of the phenotypic variance that can be attributed to common SNPs, which was estimated to be about 24%. Even though it is still controversial whether PRS is a good enough predictor for clinical use (105, 106), it may be useful to identify high-risk subjects where disease prevention studies can focus.

## Modifiable Risk Factors for AD

Observational studies have suggested that diabetes, mid-life obesity, mid-life hypertension, high cholesterol, and smoking are modifiable risk factors for AD (107). In terms of modifiable protective factors, education has been robustly shown to reduce AD risk (108). However, for many modifiable factors, no consistent pattern was found across studies (109). A recent comprehensive meta-analysis of 93 modifiable risk factors was conducted from 323 retrospective case/control and prospective cohort studies, which were selected after a systematic review of 16,906 publications (110). This study analyzed associations between AD risk and medical, dietary and occupational exposures as well as serum biochemistry, preexisting diseases, lifestyle, and psychological factors. The identified potentially protective factors include medical exposures of estrogen, statin, antihypertensive medications, and non-steroidal anti-inflammatory drugs, along with dietary exposures to folate, vitamin E/C, and coffee. Other potentially beneficial factors include a history of arthritis, heart disease, and cancer, cognitive activity, current smoking (in Western population), light-to-moderate drinking, and stress. Factors associated with increased risk were hyperhomocysteinemia, depression, frailty, carotid atherosclerosis, hypertension, low diastolic blood pressure, and low education. Evidence for metabolic factors appeared to be inconsistent. Notably, type 2 diabetes mellitus was associated with increased risk in an Asian population, but metabolic syndrome was associated with decreased risk. Moreover, both high body mass index (BMI) in mid-life and low BMI in late-life were associated with increased risk. Most recently, the Lancet Commissions estimated the population attributable fraction of the following modifiable risk factors: hearing loss (9.1%), “less education” (7.5%), followed by smoking, depression, physical inactivity, social isolation, hypertension, diabetes, and mid-life obesity in a declining order (2). The authors estimated that about 35% of total dementia risk may be attributable to a combination of these risk factors. Any preventive interventions addressing these factors can be applied independently of the presence of other factors like genetic risk. However, identifying individuals who would benefit most from a certain intervention due to their genetic risks remains an open question.

It has been widely hypothesized that factors such as physical activities that protect cardiovascular health would also protect the brain from AD and other dementias. A prospective interventional trial (111) along with observational studies (112–117) supports the notion that physical activity may reduce dementia risk. However, a recent meta-analysis of several randomized controlled trials (118) does not support the beneficial effects of long-term exercise on dementia or cognitive decline. A recent large trial with random assignment of intensive lifestyle intervention over 10 years showed that sustained relative weight loss and increases in physical activity did not alter the subsequent prevalence of cognitive impairment in diabetic and obese patients (119). It is currently uncertain whether life style intervention would prevent AD.

Another method to address the causal relationship of a potential modifiable risk factor (exposure) with an outcome such AD is MR. MR infers causation between the exposure and the outcome if the genetic variants associated with the exposure are also associated with the outcome. In other words, if a clinical risk factor P1 is causal for a disease P2, then genetic risk variants G associated with P1 would also be associated with P2 (G → P1 → P2) (120, 121). In principle, MR is expected to avoid bias from reverse causation and generally reduce confounding from other modifiable environmental exposures as it is a common problem in observational studies. Thus, it may provide relatively unbiased estimates of the effect of the modifiable risk factor being studied (122). A limitation of the MR approach is that at least one genetic variant that can reliably predict the exposure is required.

Larsson et al. (123) applied MR on genetic data from over 17,000 AD cases and over 37,000 controls to analyze the effect of 24 potentially modifiable risk factors. Assuming linear association and absence of any alternative causal pathways, genetically predicted higher educational attainment was found to significantly lower odds for AD. This finding is consistent with observational studies. Surprisingly, suggestive evidence was also found for genetically predicted higher quantity of cigarette smoking and lower odds of AD, which is inconsistent with results from cohort studies (124). In addition, genetically predicted higher 25-hydroxyvitamin D concentrations were associated with decreased AD odds, whereas higher coffee consumption with increased odds. Genetically predicted alcohol consumption, serum folate, serum vitamin B12, homocysteine, cardiometabolic factors, and C reactive proteins were not predicted to influence AD risk. One limitation of this study is that the authors used summary of association results rather than actual genotypes. Another MR study using different intermediate factors on the same set of GWAS data found that genetically predicted higher systolic blood pressure may be protective for AD (125), which is compatible with the reported protective effect of higher diastolic blood pressure (110). This result is nonetheless counterintuitive, given the known detrimental health effects of hypertension. This study also found a protective effect of genetically predicted higher smoking quantity. In addition, findings on cholesterol were not consistent with a causal effect on AD risk, after controlling for the confounding effect of *APOE*. Clearly, more research on larger datasets that include recorded clinical and lifestyle factors are needed to confirm or reject causal implications of some modifiable risk factors of AD.

In addition to the MR approach, there are other attempts to find interplay between genetic and environmental factors. An example is to study gene–environment interactions (126) and one study have shown that estrogen use may be associated with less cognitive decline among *APOE4* negative women (127).

## Current State of Development of Treatment for AD and Future Outlook

Currently, no disease modifying treatment is available for AD. The only treatments available are treating symptoms, but not the causes of the disease and its progression (128). This statement holds despite the stunning fact that between 2002 and 2014, more than 400 drug trials for AD have been performed but subsequently failed (129). More recently, several large drug trials aiming at reducing the amyloid burden had failed to show efficacy. Attempts to reduce Aβ production (130) as well as immunotherapeutic approaches to clear amyloid plaques from the brain did not show efficacy in slowing down or halting the course of AD (131, 132). Biogen’s immunotherapeutic drug Aducanumab reported positive Phase 1 results on removing brain Aβ plaques and clinical benefits (133). The result of a larger phase 3 trial is still pending.

Explanations of the failure of so many drug trials targeting Aβ argue for possible flaws in the amyloid hypothesis, or the possibility that the disease being too advanced at the time of intervention (131, 134). Drug trials in presymptomatic mutation carriers of autosomal dominant AD may shed light on whether targeting amyloid will yield any therapeutic effect (135). Ongoing drug trials include targeting anti-amyloid, anti-tau, anti-inflammatory, neuroprotection, stem cell therapy, and metabolism (136).

Advances of information technology have enabled health care providers to collect, store, and analyze large quantities of individual health data ranging from clinical information such as diagnostic test results and medication history to lifestyle factors such as smoking. At the same time, scientific community is equipped with methods to generate, process, and analyze large datasets from genomics, imaging, transcriptomics, and many other data-intensive researches. The current concept of precision medicine (137) considers clinical, behavioral, and molecular data to predict personalized disease risk, implement preventive measures, make more accurate diagnosis, and recommend treatments that maximize therapeutic effects and minimize adverse effects. To facilitate precision medicine the National Institute of Health (NIH) launched the *All of Us* research program, which plans to enroll one million participants (https://allofus.nih.gov/about/about-all-us-research-program).

Under the assumption that the treatment success of a potentially effective pharmacological intervention depends on its initiation in the presymptomatic stage, the identification of at risk subjects will be crucial to maximize treatment effect. Currently, a prevention trial as part of the Dominantly Inherited Alzheimer Network (DIAN) is under way (138). However, results from DIAN may not be representative for the majority of at risk subjects, as most AD patients do not carry Mendelian mutations. Independently, imaging amyloid and tau was shown to identify such at risk subjects (139). In reality, however, large-scale application of imaging biomarkers as a broad population screening method is difficult to implement, due to its invasiveness, high cost, and limited availability of equipment. Other fluid biomarkers have been useful in research studies (21), but their broad use in clinical settings was limited due to lack of established reproducible assays and the reluctance of patients to agree to lumbar puncture procedure (140). Most recently, reports on high-performance plasma amyloid-β biomarkers showed promising accuracy in predicting brain amyloid-β burden (141). Unlike these biomarkers, known genetic risks of a subject remain stable over time and are not influenced by any confounding factors. Currently, genetic risk factors can be assessed at a very low cost starting at around $50 per sample for array-based genotyping data. These arrays cover common variants that may include disease risk variants, which can be further used to impute additional disease risk variants. Moreover, the cost for more comprehensive WES and WGS is down trending toward several hundred dollars. Thus, it is feasible that genetic risk profiles may be used alone or combined with other biomarkers to select at risk subjects in preclinical stage for closer follow-ups and enrollment into preventive studies.

Genetic testing may also increase diagnostic precision in patients with dementia. A recent study showed that known pathogenic mutations for AD and frontotemporal dementia were found with similar proportion in familial LOAD and sporadic LOAD patients. Mutations for Parkinson’s diseases (PD) and amyotrophic lateral sclerosis were also found in clinically diagnosed AD subjects (86). Therefore, genetic testing may prevent other neurodegenerative diseases, which may even have some treatment options, from being misdiagnosed as AD. Combined with fluid and imaging biomarkers, genetics may further increase diagnostic accuracy to ensure clinical trials are done in truly AD patients. Furthermore, instead of treating AD as a homogeneous disease, genetics and other diagnostic methods hold the potential to identify functional disease subtypes that could be specifically targeted.

Another advantage of genetic screening, especially in subjects with family history of dementia, would be the improved risk assessment. An accurate risk assessment could lead to specific consultation for preventive measures addressing modifiable risk factors, such as early use of hearing aids and managing metabolic symptoms. Linking genomic data and electronic health record (EHR) may further help researchers to identify how genetic factors interact with other health conditions such as the impact of medication use on disease risk. For example, an EHR-based analysis found that salbutamol, a β2-adrenoreceptor agonist often prescribed for asthma, is associated with a 34% lower risk of PD and propranolol, a drug frequently prescribed for hypertension, with increased risk (142). Similar approaches of EHR mining may discover medications that alter AD risk. Genetic risk factors had strongly supported a role of immune pathways in AD. Analysis of large EHR data could find out whether drugs that target the immune system had an impact on risk for AD.

Large-scale genetic testing may come from consumer genetic services as they become more broadly available. More than three million people already had their DNA tested at 23&Me and received their carrier status of *APOE4* among other risk variants affecting health. Currently, there are hundreds of companies offering similar services and the list is growing (143). The number of people equipped with personal genetic data will likely continue to increase in the general population. Such consumer genetic data may be integrated into EHR to assist diagnostic assessments and choice of treatment. For example, clinicians may consider avoiding propranolol and other β-blockers for patients with genetic predisposition for PD.

In addition to risk variants, genetic studies will identify more protective variants against AD. As the sample size becomes larger, researchers may identify potentially protective factors in subjects who carry strong risk factors such as homozygosity of *APOE4*, but do not develop AD at an advanced age (65). Identification of protective variants in such a population may lead to possible new drugs that act through a similar mechanism. A recent example for protective genetic variants fueling new effective therapeutics was the development of PCSK9 inhibitor for hypercholesterolemia (144, 145). The newly identified gene *PLCG2* that harbors rare protective variants is highly expressed in microglia and may be a target to be exploited for drug discovery in AD (81). Certainly, a hope is that ongoing sequencing efforts (146) would identify more protective variants that can be targeted by drugs.

**Figure 3:**
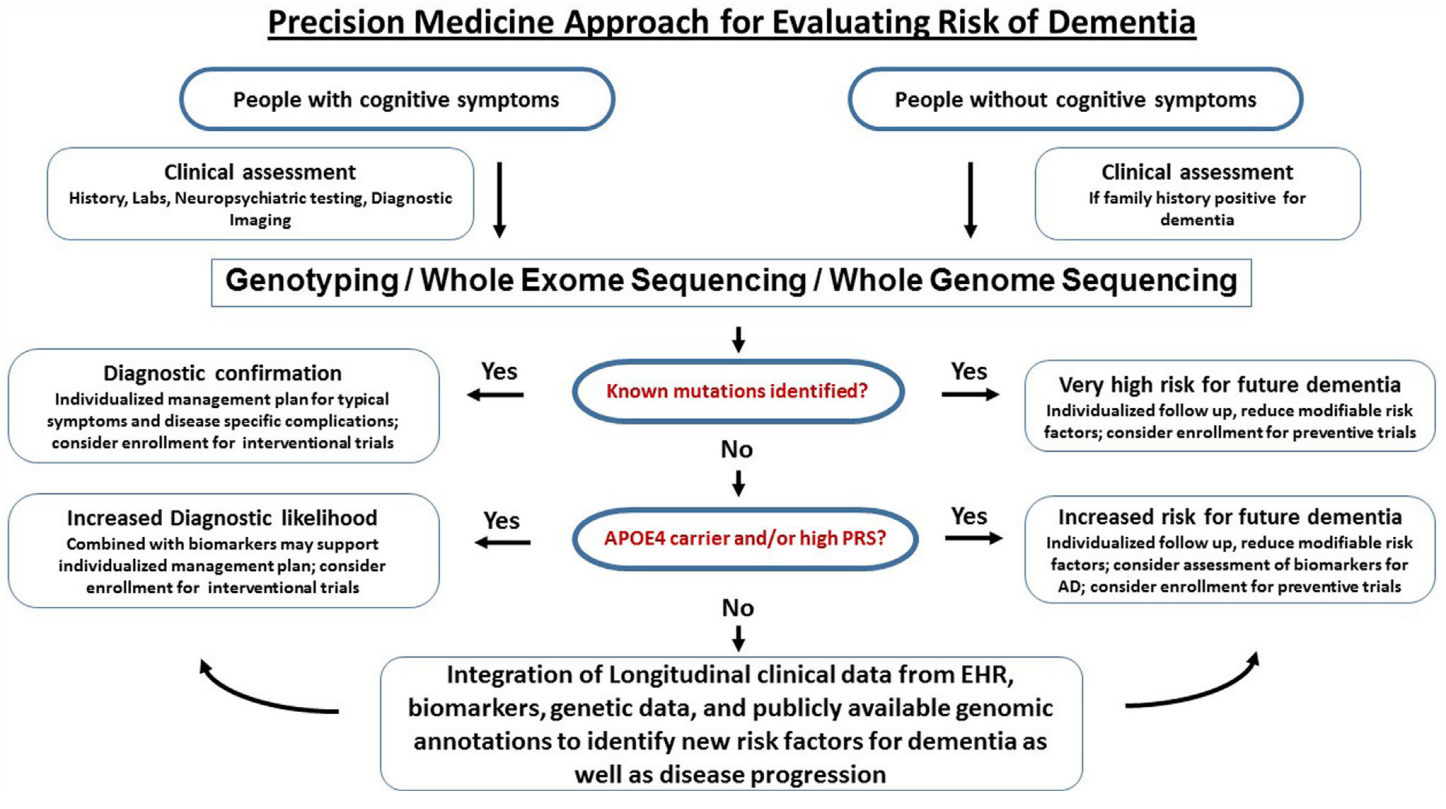
Precision medicine approach for dementia. This is a graphical outline of how genetic and genomic information could be combined and integrated with electronic health records (EHRs) to improve the accuracy of dementia diagnosis and facilitate drug discovery. Middle-aged and older people (e.g., age > 50) are enrolled in an ongoing protocol that includes medical and family history, diagnostic assessment, and access to EHR. For those who have signs of cognitive impairment, genetic testing using either mutation-panels, genotyping arrays, whole exome sequencing or whole genome sequencing depending on the clinical question is performed alongside biomarkers. If a dementia diagnosis is confirmed through genetics and biomarkers, the patients are referred to specialized behavioral and pharmacological intervention and have the option to participate in drug trials. For the majority of subjects who do not have definitive biological findings, a likelihood risk score may be estimated based on the genetic and biomarker profiles. These risk scores may provide support for clinical diagnosis and identify subjects at risk for dementia. The presymptomatic at risk subject may be enrolled in longitudinal studies on prevention and those who never develop dementia despite having high risk may be studied to identify protective factors.

A workflow for clinical translational research implementing clinical assessments, genetics, and biomarkers into clinical research (as discussed above) is graphically described in Figure 3. Of course, large-scale population level genetic testing also brings ethical challenges. Clinicians and researchers need to take into account the respective guidelines for genetic testing (147). Current studies indicate that the majority of individuals tested for autosomal dominant forms of AD under a standardized counseling protocol demonstrated effective coping skills. Negative psychological reactions were absent after several months and the testing was perceived to be beneficial. The potential benefits, harms, and dilemmas of genetic testing and impacts on family members were detailed in a case report (148). If results of risk factors are returned to the participants, counseling needs to be provided and psychosocial support should be made available. It is important that patients and customers of consumer genetics services understand that typical risk factors are not deterministic for AD. The ethical, legal, and social implications of genetic testing such as testing-induced harm and discrimination are an active area of research at NIH (149).

In summary, the current approach for AD consists of optimizing modifiable risk factors to reduce and delay symptom onset as well as symptomatic treatment after disease onset. The dawn of the big data era may make it feasible to advance precision medicine by systematically integrating massive biological data generated by next-generation genomic sequencing, biomarker testing, and EHRs. This development is likely to shed more light to the complex biology of AD and accelerate development of better prevention, diagnosis, and treatments.

## Author Contributions

YF-H contributed to conception of the work, data collection, literature research, data interpretation, drafting of the article, critical revision, and final approval of the version to be published. WL contributed to data interpretation, drafting of the article, and critical revision. PD contributed to conception of the work, critical revision, and final approval of the version to be published.

## Conflict of Interest Statement

The authors declare that the research was conducted in the absence of any commercial or financial relationships that could be construed as a potential conflict of interest.

## Abbreviations

AA, Alzheimer’s Association; Aβ, amyloid beta; AD, Alzheimer’s disease; BMI, body mass index; CSF, cerebrospinal fluid; DIAN, Dominantly Inherited Alzheimer Network; EOAD, early onset Alzheimer’s disease; EHR, electronic health record; FDG, 18-fluoro-deoxyglucose; GWAS, genome-wide association studies; LOAD, late onset Alzheimer’s disease; MR, Mendelian randomization; NFT, neurofibrillary tangles; NHGRI-EBI, National Human Genome Research Institute-European Bioinformatics Institute; NIH, National Institute of Health; NIA, National Institute on Aging; PD, Parkinson’s diseases; PET, positron emission tomography; PI, physical interaction; PIB, Pittsburgh compound B; PRS, polygenic risks score; SNP, single-nucleotide polymorphism; WES, whole exome sequencing; WGS, whole genome sequencing.
